# Cardiovascular Malformations in CHARGE Syndrome with DiGeorge Phenotype: Two Case Reports

**DOI:** 10.1155/2016/8013530

**Published:** 2016-11-10

**Authors:** Kazushi Yasuda, Eiji Morihana, Naoki Fusazaki, Shiro Ishikawa

**Affiliations:** ^1^Department of Neonatal Cardiology, Fukuoka Children's Hospital, Fukuoka, Japan; ^2^Department of Pediatric Cardiology, Fukuoka Children's Hospital, Fukuoka, Japan

## Abstract

Both CHARGE syndrome and DiGeorge anomaly are frequently accompanied by cardiovascular malformations. Some specific cardiovascular malformations such as interrupted aortic arch type B and truncus arteriosus are frequently associated with 22q11.2 deletion syndrome, while conotruncal defects and atrioventricular septal defects are overrepresented in patients with CHARGE syndrome.* CHD7* gene mutation is identified in approximately two-thirds of patients with CHARGE syndrome, and chromosomal microdeletion at 22q11.2 is found in more than 95% of patients with 22q11.2 deletion syndrome. CHARGE syndrome is occasionally accompanied by DiGeorge phenotype. We report two patients with dysmorphic features of both CHARGE syndrome and 22q11.2 deletion syndrome. Although both of the two cases did not have 22q11.2 deletion, they had typical dysmorphic features of 22q11.2 deletion syndrome including cardiovascular malformations such as interrupted aortic arch type B. They also had characteristic features of CHARGE syndrome including ear malformation, genital hypoplasia, limb malformation, and endocrinological disorders.* CHD7* gene mutation was confirmed in one of the two cases. When a patient with cardiovascular malformations frequently associated with 22q11.2 deletion syndrome does not have 22q11.2 deletion, we suggest that associated malformations characteristic of CHARGE syndrome should be searched for.

## 1. Introduction

CHARGE syndrome is a pleiotropic disorder, the name of which is derived from the acronym summarizing its six clinical features: ocular coloboma, heart defects, choanal atresia, retardation of growth/development, genital anomalies, and ear anomalies/deafness.* CHD7* is the only gene mutation of which is known to cause CHARGE syndrome, and the mutation is identified in approximately two-thirds of patients with a clinical diagnosis of CHARGE syndrome [1].

DiGeorge anomaly is characterized by thymic deficiency, hypoparathyroidism, and cardiovascular malformation, and it is now recognized that 22q11.2 deletion syndrome encompasses the phenotypes previously described as DiGeorge anomaly [2]. Microdeletion of chromosomal 22q11.2 is found in more than 95% of patients with 22q11.2 deletion syndrome.* TBX1*, a gene located within the A-B region of chromosome 22q11.2, is responsible for the phenotypic features seen in this syndrome [3].

The clinical overlap between CHARGE syndrome and 22q11.2 deletion syndrome is recently considered to be more common than anticipated [4]. We here report two patients with dysmorphic features of both CHARGE syndrome and 22q11.2 deletion syndrome and especially describe their cardiovascular malformations.

## 2. Case Presentation

**Case 1 .**

A male fetus was diagnosed as having heart defects and hydramnios at the fetal age of 32 weeks. He was born after 38 weeks of gestation, weighing 2448 g. Shortly after birth, he was admitted to the neonatal intensive care unit and diagnosed as having truncus arteriosus and interrupted aortic arch. Prostaglandin E_1_ was administered intravenously to maintain the arterial duct patency. Calcium preparation was given because of hypocalcemia, which was subsequently confirmed to be due to hypoparathyroidism.

**Figure 1:**
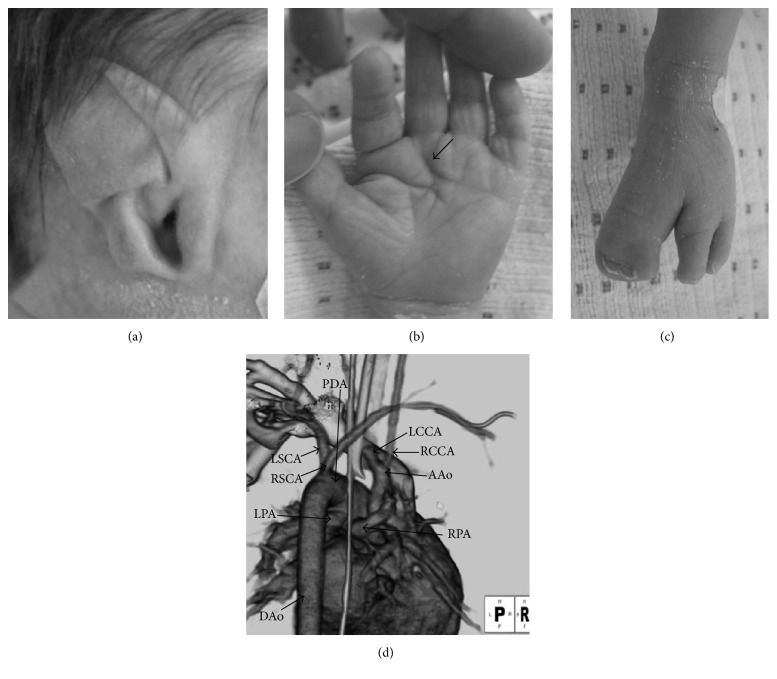
Dysmorphic features and cardiovascular malformations in Case 1. (a) “CHARGE ear.” (b) Hockey-stick palmar crease (arrow). (c) Cleft foot. (d) 3D-CT angiography showed truncus arteriosus type A4, interrupted aortic arch type B, and aberrant origin of the right subclavian artery. AAo, ascending aorta; DAo, descending aorta; LCCA, left common carotid artery; LPA, left pulmonary artery; LSCA, left subclavian artery; PDA, patent ductus arteriosus; RCCA, right common carotid artery; RPA, right pulmonary artery; RSCA, right subclavian artery.

After transfer to our hospital at 9 days of age, he was diagnosed as having truncus arteriosus type A4, interrupted aortic arch type B, ventricular septal defect, and aberrant origin of the right subclavian artery. He had many dysmorphic features, such as ocular coloboma, choanal atresia, ear anomaly, hearing impairment, thymic deficiency, cleft foot, cleft hand, hockey-stick palmar crease, and distinctive facial features which are square face with broad prominent forehead, “hooded eyelids,” ocular hypertelorism, and micrognathia (Figure 1). He seemed to have both cardiovascular malformations frequently associated with 22q11.2 deletion syndrome and features seen in CHARGE syndrome and/or 22q11.2 deletion syndrome. Fluorescence in situ hybridization analysis did not demonstrate chromosomal microdeletion at 22q11.2, but* CHD7* mutation was subsequently confirmed (Table 1). His parents and siblings did not have any chromosomal disorders and anomalies.

At 14 days, he underwent bilateral pulmonary artery banding. Thymic deficiency was confirmed intraoperatively. Flow cytometry of peripheral blood revealed cellular immunodeficiency. A diagnosis of complete DiGeorge anomaly was made, and antimicrobial agents and immunoglobulin were administered.

He was transferred back to the previous hospital for treatment for immunodeficiency at 30 days and underwent cord blood transplantation at 4 months [5]. After successful engraftment, he underwent stent placement for the arterial duct at 10 months.

**Case 2 .**

A male neonate was born at the gestational age of 39 weeks, weighing 2794 g. After birth, he was diagnosed as having transient tachypnea of the newborn based on the occurrence of tachypnea and cyanosis. He was also suspected to have hypoplastic aortic arch and hypoplastic left ventricle.

He was referred to our hospital at 6 days of age and diagnosed as having double outlet right ventricle, subpulmonary ventricular septal defect, interrupted aortic arch type B, subaortic stenosis, hypoplastic ascending aorta, aberrant origin of the right subclavian artery, and restrictive atrial septal defect. At 15 days, balloon atrial septostomy was performed. Computed tomography showed additional anomalies of cephalocervical arteries, that is, absence of the right internal carotid artery and the right posterior communicating artery, and aberrant origin of the right external carotid artery from the right subclavian artery and the right vertebral artery directly from the ascending aorta.

**Figure 2:**
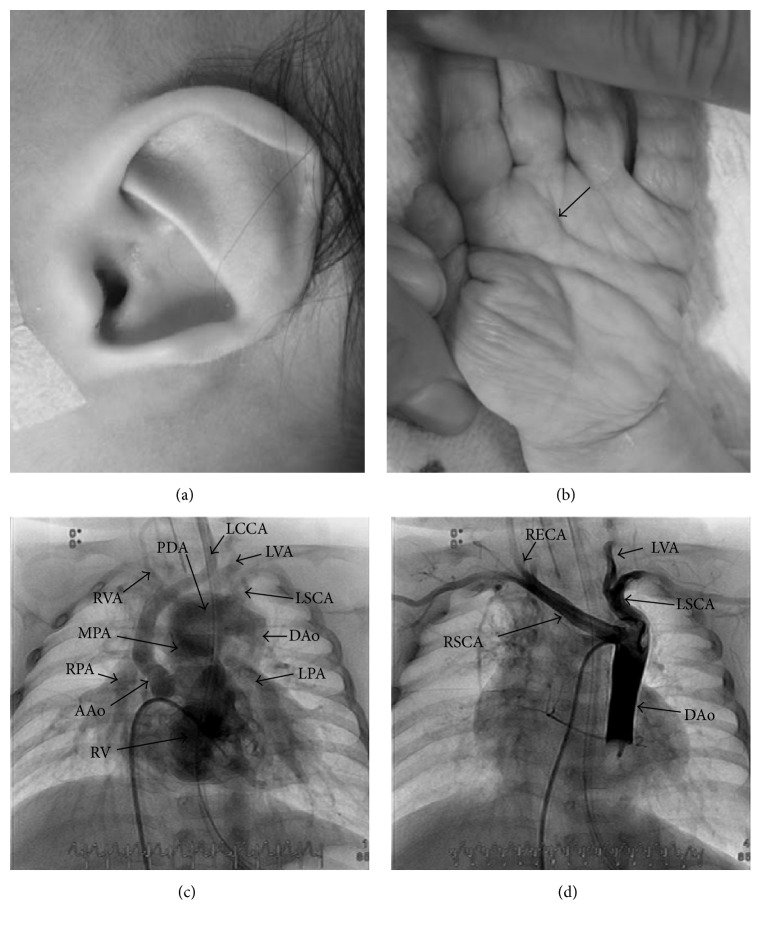
Dysmorphic features and cardiovascular malformations in Case 2. (a) “CHARGE ear.” (b) Hockey-stick palmar crease (arrow). (c) Right ventriculography demonstrated double outlet right ventricle, interrupted aortic arch type B, and hypoplastic ascending aorta. (d) Descending aortography with balloon occlusion. Note aberrant origin of the right subclavian artery. AAo, ascending aorta; DAo, descending aorta; LCCA, left common carotid artery; LPA, left pulmonary artery; LSCA, left subclavian artery; LVA, left vertebral artery; MPA, main pulmonary artery; PDA, patent ductus arteriosus; RECA, right external carotid artery; RPA, right pulmonary artery; RSCA, right subclavian artery; RV, right ventricle; RVA, right vertebral artery.

At 16 days, he underwent bilateral pulmonary artery banding. No thymus was found intraoperatively, but cellular immunocompetence was subsequently confirmed. He had many other endocrinological disorders and dysmorphic features, that is, hypothyroidism, hypoparathyroidism, ear malformation, hearing impairment, micropenis, cryptorchidism, hockey-stick palmar crease, and distinctive facial features such as square face with broad prominent forehead and prominent nasal bridge (Figure 2). He also seemed to have both cardiovascular malformations frequently associated with 22q11.2 deletion syndrome and many features seen in CHARGE syndrome and/or 22q11.2 deletion syndrome. Fluorescence in situ hybridization analysis did not demonstrate chromosomal microdeletion at 22q11.2 in this patient as well (Table 1).* CHD7* gene mutation was not examined. His parents and siblings did not have any chromosomal disorders and anomalies.

At 3 months, he underwent biventricular repair by modified Norwood operation and Rastelli operation, and administration of prostaglandin E_1_ was discontinued.

## 3. Discussion

22q11.2 deletion syndrome is frequently accompanied by cardiovascular malformations such as conotruncal anomalies (tetralogy of Fallot, truncus arteriosus), interrupted aortic arch, right aortic arch, and aberrant origin of the subclavian artery [6]. In a previous large series, 43% and 34% of patients with 22q11.2 deletion syndrome were found to have interrupted aortic arch type B and truncus arteriosus, respectively. Conversely, 68% of patients with interrupted aortic arch type B and 33% of patients with truncus arteriosus had 22q11.2 deletion syndrome [7]. Therefore, the characteristic pattern of cardiovascular malformations in addition to hypocalcemia and absence of the thymus is likely to lead to early clinical diagnosis of 22q11.2 deletion syndrome.

CHARGE syndrome shows a variety of the combination and extent of symptoms and findings. Immediate evaluations of the airway, feeding, heart, and hearing are required, because multiple life-threatening medical conditions are often found in CHARGE syndrome. Cardiovascular malformations are present in 75% to 85% of individuals with CHARGE syndrome [8]. The severity and spectrum of congenital heart defects had been thought to vary [9]. It is recently reported that conotruncal defects and atrioventricular septal defects are overrepresented in patients with* CHD7* mutations compared with patients with nonsyndromic heart defects [10]. Tetralogy of Fallot was relatively frequent among the conotruncal defects, whereas interrupted aortic arch, double outlet right ventricle, and truncus arteriosus were less frequently seen. In contrast to 22q11.2 deletion syndrome, the pattern of cardiovascular malformations is less likely to lead to the diagnosis of CHARGE syndrome.

CHARGE syndrome is rarely accompanied by DiGeorge phenotype without chromosome 22q11.2 deletion [8]. It has long been recognized that CHARGE syndrome and 22q11.2 deletion syndrome have overlapping phenotypic features [11]. The phenotypic similarity between CHARGE syndrome and 22q11.2 deletion syndrome is also apparent in mice with haploinsufficiency of* CHD7* and* TBX1*. Both genes are shown to interact and are required in pharyngeal ectoderm for fourth pharyngeal arch artery development. In addition, both genes are important in development of the thymus and semicircular canals [12, 13].

de Lonlay-Debeney et al. reported that five patients presented with features of both CHARGE syndrome and 22q11.2 deletion syndrome [14]. Four of the five patients had cardiovascular malformations, that is, tetralogy of Fallot, truncus arteriosus, aberrant origin of the left subclavian artery with right aortic arch, and hypoplastic aortic arch with ventricular septal defect, respectively. None of the five patients had chromosome 22q11.2 deletion, similarly to our cases. Recently, Corsten-Janssen and colleagues reported that five patients with a clinical diagnosis of CHARGE syndrome and a proven 22q11.2 deletion were identified [4]. However, 25 of 33 patients with typical 22q11.2 deletion features and a* CHD7* mutation had no 22q11.2 deletion. In addition, 5 of 20 patients who were clinically suspected of 22q11.2 deletion syndrome but with neither a 22q11.2 deletion nor a* TBX1* gene mutation had a* CHD7* mutation. Although the clinical overlap between CHARGE syndrome and 22q11.2 deletion syndrome is recently considered to be more common than anticipated, such overlapping cases seem to be unlikely to have a 22q11.2 deletion, regardless of the high probability of the chromosomal microdeletion in 22q11.2 deletion syndrome.

When a patient with cardiovascular malformations frequently associated with 22q11.2 deletion syndrome does not have 22q11.2 deletion, we suggest that associated malformations characteristic of CHARGE syndrome should be searched for.

## Figures and Tables

**Table 1:** Clinical features and laboratory findings of the present cases. Cases 1 and 2 were diagnosed as definite and probable/possible CHARGE syndrome, respectively. Cardiovascular malformations frequently associated with 22q11.2 deletion syndrome and characteristics^*∗*^ originally described as DiGeorge anomaly [2] were also found in both cases, but fluorescence in situ hybridization analysis did not demonstrate chromosomal microdeletion at 22q11.2. See the text for details of cardiovascular malformations. Clinical diagnosis of CHARGE syndrome was based on Blake's criteria [8]. N/e, not examined.

Features/findings	Case 1	Case 2	Frequency in CHARGE syndrome [1, 8]	Frequency in 22q11.2 deletion syndrome [2, 3]
Major diagnostic characteristics for CHARGE syndrome				
Ocular coloboma	Yes	No	80–90%	
Choanal atresia or stenosis	Yes	No	50–60%	
Cranial nerve dysfunction or anomaly				
I: hyposmia or anosmia	N/e	N/e	70–90%	
VII: facial palsy	No	No		
VIII: auditory nerve hypoplasia	Yes	Yes		
IX/X: swallowing problems	Yes	Yes		
Characteristic CHARGE syndrome ear	Yes	Yes	90%	
Minor diagnostic characteristics for CHARGE syndrome				
Genital hypoplasia (micropenis, cryptorchidism)	No	Yes	70–80%	
Developmental delay (delayed milestone, hypotonia)	Yes	Yes	100%	75%
Cardiovascular malformation^*∗*^	Yes	Yes	75–85%	49–83%
Growth deficiency	Yes	Yes	70%	4%
Orofacial cleft	No	No	15–20%	9–11%
Tracheoesophageal fistula	No	No	15–20%	
Distinctive face	Yes	Yes	70–80%	
Others				
Immune deficiency/thymic deficiency^*∗*^	Yes	Yes	Rare	Most
Hypoparathyroidism^*∗*^/hypocalcemia	Yes	Yes	Rare	17–60%
Hypothyroidism	No	Yes		
Hand anomalies	Yes	Yes	50%	
*CHD7* gene mutation	Yes	N/e	67%	
Chromosome 22q11.2 deletion	No	No		96%
Diagnostic criteria for CHARGE syndrome [8]	4 major + 4 minor	2 major + 5 minor		
	Definite	Probable/possible		
Characteristics^*∗*^ originally described as DiGeorge anomaly [2]	Yes	Yes		
